# A rare compound heterozygous *EIF2AK4* mutation in pulmonary veno-occlusive disease

**DOI:** 10.1186/s12890-022-02256-9

**Published:** 2022-11-30

**Authors:** Chun Zhang, Qiang Du, Sha Wang, Ruifeng Zhang

**Affiliations:** 1 Department of Respiratory Medicine, Zhongda Hospital of Southeast University, Dingjiaqiao 87, Nanjing City, Jiangsu Province People’s Republic of China; 2 DIAN Diagnostics, Hangzhou, People’s Republic of China

**Keywords:** PVOD, *EIF2AK4*, Compound heterozygous mutation, Whole-exome sequencing

## Abstract

**Background:**

Pulmonary veno-occlusive disease (PVOD) is a rare, progressive, and oft-fatal condition of pulmonary arterial hypertension that is typically difficult to diagnose and treat. However, with the development of next-generation sequencing technology, an increasing number of patients with PVOD are being diagnosed.

**Methods:**

Initially, we used whole exome sequencing (WES) to identify the proband as a rare compound heterozygous mutation of *EIF2AK4* in PVOD. Subsequently, the parents of patient underwent *EIF2AK4* screening by Sanger sequencing.

**Results:**

In this study, we describe the family tree of a patient with PVOD with a rare compound heterozygous *EIF2AK4* mutation. Moreover, we identified a new *EIF2AK4* mutation, c.2236_2237insAAGTCCTTCT, in exon 12 of the proband and his mother. This frameshift mutation led to premature termination of the coding protein sequence and widespread loss of protein function, which promoted the development of PVOD.

**Conclusions:**

Our results expand our understanding of the *EIF2AK4* mutation spectrum in patients with PVOD, as well as highlight the clinical applicability of WES.

## Background

Pulmonary veno-occlusive disease (PVOD) is a sporadic or familial hereditary disease; however, its true prevalence and incidence in the general population are not fully understood. The prevalence of PVOD is conservatively estimated at 1–2 per million population, and the annual incidence of primary and hereditary PVOD is estimated to be 0.1–0.5 million, which is likely underestimated given the difficulty in diagnosis [1]. Studies have shown that the mortality rate of PVOD is approximately 72% within 1 year following diagnosis, and the mean time from the onset of initial symptoms to death or lung transplantation is 24.4 months [1]. According to the 2015 ESC/ERS guidelines, the presence of a biallelic *EIF2AK4* mutation is sufficient to confirm the diagnosis of PVOD without additional histological confirmation [2]. In this study, we described the family tree of a patient with PVOD with a rare compound heterozygous *EIF2AK4* mutation, who exhibited a good response to targeted therapeutics, different from most patients with PVOD. We speculate that the true prevalence and incidence of PVOD in the general population is even greater than anticipated. With the development of next-generation sequencing (NGS) technology, an increasing number of patients with PVOD are being diagnosed.

## Methods

### Ethical approval and consent

The study protocol was approved by the Ethics Committee of Zhongda Hospital affiliated with Southeast University (Approval No: 2022ZDSYLL062-Y01) and adhered to the Declaration of Helsinki. The patients included in this study underwent genetic counseling and signed written informed consent for their data and samples to be used for research purposes.

### Subjects and clinical characteristics

The proband was diagnosed according to current guidelines, including right heart catheterization, which showed severe PAH with a mean pulmonary artery pressure of 76 mmHg and a pulmonary arterial wedge pressure of 14 mmHg. Computed angiography pulmonary angiography revealed negative findings for pulmonary embolism but revealed a ground-glass shadow and a markedly dilated main pulmonary artery branch. Samples from the proband and his parents were sent for genetic diagnostic testing to DIAN Diagnostics Corporation, Hangzhou, China. The proband was screened for *EIF2AK4* mutations, and two biallelic *EIF2AK4* mutation sites were found, which confirmed the final diagnosis of PVOD.

### Whole exome sequencing

Genomic DNA from the proband was extracted from peripheral blood for genetic analysis. Whole exome sequencing of the proband was performed by DIAN Diagnostics Corporation, Hangzhou, China, using the IDT xGen Exome Research Panel v1.0 with a paired-end read length of 150 bp. Sequencing was performed using a NovaSeq 6000 system (Illumina). The average sequencing depth was 100× and the average genome coverage was 99.59%. Sequences were aligned to the human reference genome hg19 and variants were called using the Genome Analysis Toolkit and annotated using VEP and Snpeff. The data interpretation rules mainly refer to the guidelines of the American College of Medical Genetics and Genomics. The variants were classified into five categories according to the recommendations of the American College of Medical Genetics and Genomics: benign, likely benign, unknown significance, likely pathogenic, and pathogenic.

### Sanger sequencing

Familial variants were identified by Sanger sequencing in the proband’s parents (ABI Genetic Analyzer 3130xl; Applied Biosystems, USA). RNA was extracted using standard protocols (RNeasy Mini Kit; Qiagen). Complementary DNA was transcribed using Superscript II reverse transcriptase (Life Technologies, USA) with random hexamer primers (Roche Diagnostics Deutschland GmbH, Germany). Polymerase chain reactions were designed using primers located in exons adjacent to the predicted alternative or lost splice sites. The sizes of the amplified products were measured using agarose gel electrophoresis and Sanger sequencing.

## Results

**Fig. 1:**
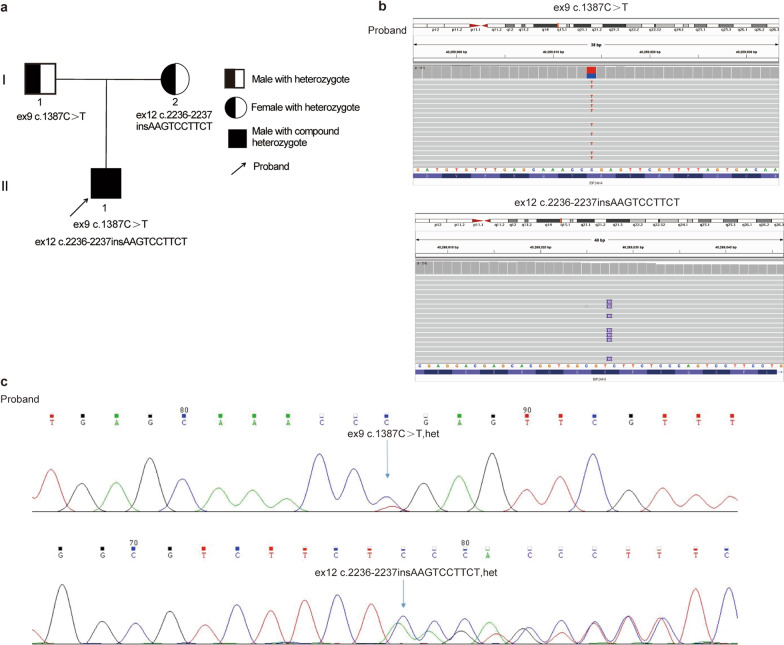
Family tree and genetic characteristics of the proband in PVOD. **a** Pedigree of the family with a rare compound heterozygous *EIF2AK4* mutation in PVOD. **b** Visualization of mutations in *EIF2AK4* using Integrative Genomics Viewer. The proband was a compound heterozygote with mutations in exon 9 c.1387C > T and exon 12 c.2236_2237insAAGTCCTTCT. **c** WES showed that variations in c.1387C > T and c.2236_2237insAAGTCCTTCT were heterozygous in the proband. Blue arrows indicate the point mutation. PVOD, Pulmonary veno-occlusive disease; WES, whole exome sequencing

In 2021, our group reported two cases of PVOD that were initially misdiagnosed as idiopathic pulmonary arterial hypertension owing to the lack of typical findings [3]. Both patients responded well to PAH-targeted therapy. Subsequently, we evaluated the genetic background of the first proband’s parents in the present study. Although the proband’s father and mother had no associated lung symptoms or disease history, heterozygous mutations in *EIF2AK4* were confirmed by Sanger sequencing. Moreover, a new *EIF2AK4* mutation, c.2236_2237insAAGTCCTTCT, in exon 12, was also found. A family tree of the patient with PVOD with a rare compound heterozygous *EIF2AK4* mutation is described in the present study. Notably, the proband had no siblings (Fig. 1a).

**Fig. 2:**
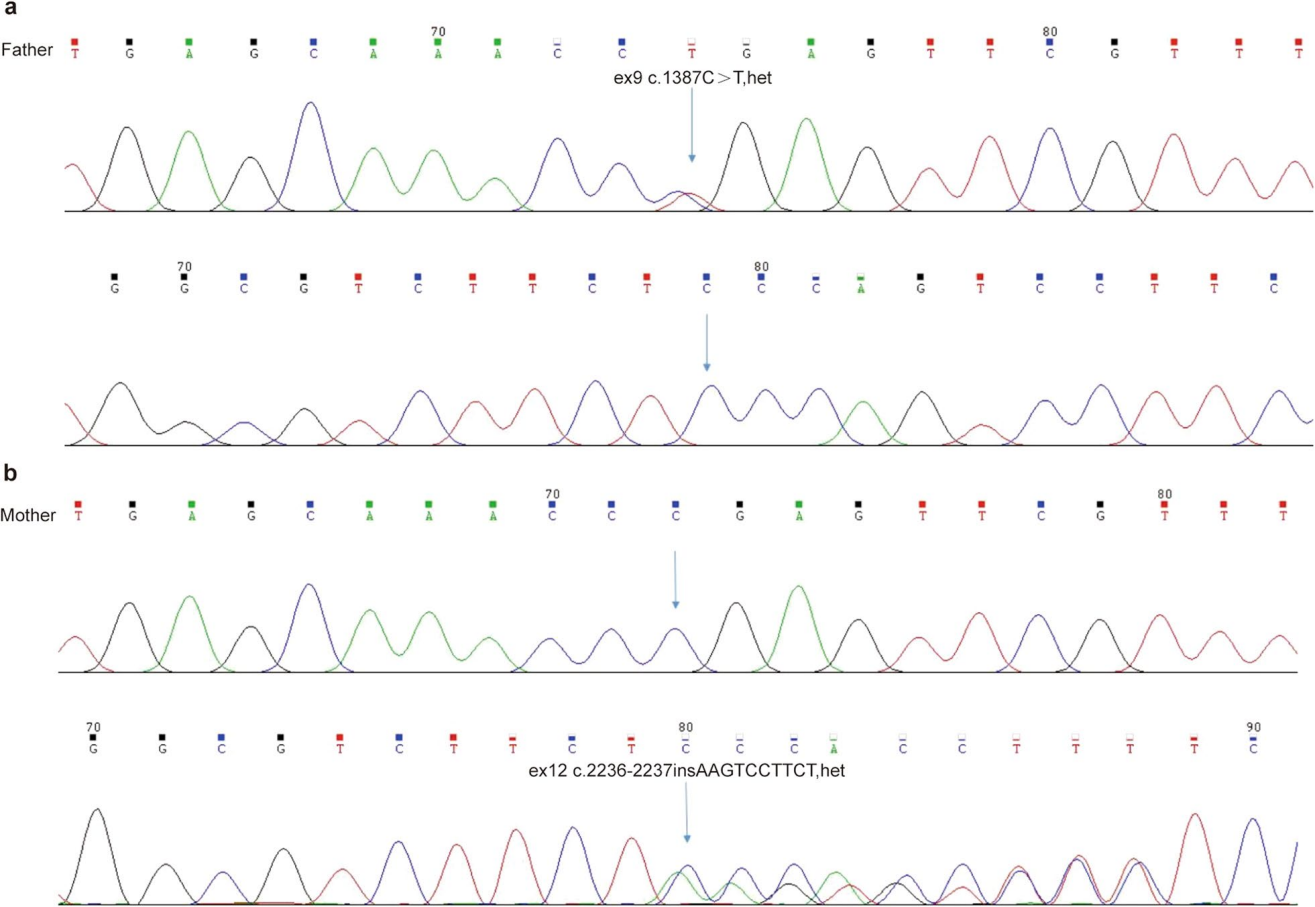
Results of *EIF2AK4* heterozygous mutations in the proband’s parents by fixed-point Sanger sequencing. **a** The father carried a heterozygous mutation in ex9 c.1387C > T. **b** The mother carried a heterozygous mutation in ex12 c.2236_2237insAAGTCCTTCT. Blue arrows indicate the point mutation

Next-generation sequencing of the whole exome was performed on the blood samples of the proband, which identified the proband as a rare compound heterozygous mutation of *EIF2AK4* in PVOD (Fig. 1b, c). The mutation had a c.1387C > T nonsense mutation in exon 9, resulting in p.Arg463*, which converts the 463 arginine-converting codon to a stop codon, whereby the coding protein sequence terminates in advance, resulting in a truncated protein or degradation. According to the Genome Aggregation Database (gnomAD) the frequency of this locus is 0% in normal East Asian populations. According to the ACMG guidelines, the evidence items of *EIF2AK4* c.1387C > T p.Arg463* were PVS1 + PM2 + PM3, and the final verdict was P, which was the pathogenic variation. In exon 12, the frameshift mutation c.2236_2237insAAGTCCTTCT results in p.Ser746*, which changes codon 746 from serine to a stop codon, resulting in a truncated protein or degradation. However, the frequency of this site in the normal East Asian population has not yet been reported. According to the ACMG guidelines, the evidence items of *EIF2AK4* c.2236_2237insAAGTCCTTCT p.Ser746* were PVS1 + PM2, and it was ultimately judged as LP, which was suspected pathogenic variation. To better understand the proband’s family pedigree, we confirmed *EIF2AK4* heterozygous mutations in the proband’s parents using fixed-point Sanger sequencing. His father carried a heterozygous mutation in ex9 c.1387C > T, whereas his mother carried a heterozygous mutation in ex12 c.2236_2237insAAGTCCTTCT (Fig. 2a, b). Combined with the family pedigree, we can infer that p.Arg463* is inherited from the proband’s father and p.Ser746* is inherited from his mother, consistent with the common autosomal recessive inheritance pattern. By referring to domestic and foreign literature and the human gene mutation database, the c.1387C > T (p.Arg463*) mutation has been found to be homozygous or compound heterozygous in patients with autosomal recessive pulmonary hypertension [4]. However, to our knowledge, the c.2236_2237insAAGTCCTTCT (p.Ser746*) mutation has not yet been reported in any public genome database.

## Discussion

The etiology of PVOD remains unknown. Previous reports have suggested that, although PVOD has no obvious genetic predisposition, it has a significant genetic background [1, 4].

Although the presence of a biallelic *EIF2AK4* mutation is sufficient to confirm the diagnosis of PVOD without additional histological confirmation [2], the precise role of *EIF2AK4* mutations in the pathogenesis of PVOD remains unclear. Zeng et al. reported that a novel *EIF2AK4* mutation, [c.4833_4836dup (p.Q1613Kfs*10)], predicted an aggressive PVOD phenotype [5]. However, Li et al. described a good response to PAH-targeted drugs in a patient with PVOD carrying the biallelic *EIF2AK4* mutation [c.1392delT(p.Arg465fs)] [6]. These controversies suggest that the development of PVOD varies greatly among individuals, and that patients may respond differently to targeted drugs. However, whether this is related to different *EIF2AK4* mutation sites in PVOD remains to be further studied.

In our case, the proband was screened for an *EIF2AK4* compound heterozygous mutation using WES, which confirmed the final diagnosis of PVOD. Moreover, we detected *EIF2AK4* heterozygous mutations in the proband’s parents by fixed-point Sanger sequencing and found a new *EIF2AK4* mutation in exon 12 of the patient and his mother. Previous studies have argued that mutations in *EIF2AK4* lead to either a defective protein or the absence of protein expression [7, 8]. The general control nonderepressible 2 (GCN2) protein, a serine-threonine kinase encoded by *EIF2AK4*, has been recently identified as a susceptibility factor driving the etiology of PVOD [2, 7]. Nossent et al. reported that pulmonary artery remodeling and decreased GCN2 expression were the common denominators in all cases of PVOD and revealed a preponderant venous remodeling in young *EIF2AK4* mutation carriers as compared with elder non-carriers [7]. In our case, the patient was a young man who responded well to PAH-targeted drugs, unlike in previous studies, which may be related to pulmonary vascular cell proliferation and microvascular remodeling associated with the *EIF2AK4* biallelic dysfunction mutation. However, the pathophysiological association between biallelic *EIF2AK4* loss-of-function mutations and pulmonary vascular cell proliferation and remodeling remains unclear. To date, no functional studies have assessed the effects of nonsense or frameshift mutations and their truncated protein products on the activity of the encoded *EIF2AK4* protein [9]. The absence of a molecular mechanism that relates the abrogated function of the protein to the phenotype remains a major hurdle in our understanding of the disease [8].

Although the typical pathological changes in PVOD are theoretically easy to understand, lung biopsy specimens of these patients are rare in actual work. Because of the rapidly progressing clinical course and often uncertain diagnosis of PVOD, rapid diagnosis and possible lung transplantation are paramount for survival [10]. Fortunately, with the advancement of technology, WES is widely used to understand the genetic etiology of a disease. An increasing number of PAH centers offer genetic testing and counseling services [11]. Compared to conventional Sanger sequencing, WES is an effective alternative method for genetic diagnosis. The clinical utility of WES will be significantly promoted in the near future with reduction in costs. In the future, we should strengthen the role of NGS in improving the diagnostic efficiency of patients with PVOD with no obvious clinical triad to achieve early detection, diagnosis, and treatment.

Our study has certain limitations. First, we did not have a complete family tree because some family members refused to undergo genetic analysis. In addition, a lung biopsy was not conducted in the proband owing to safety and economic issues. However, current guidelines indicate that heritable PVOD can be diagnosed by identifying pathogenic biallelic *EIF2AK4* variants. Last but not the least, the absence of a molecular mechanism that relates the abrogated function of the protein to the phenotype is still a major hurdle in our understanding of the disease.

## Conclusions

In summary, we describe a rare case of PVOD in which the proband had an *EIF2AK4* compound heterozygous mutation with heterozygous parents. We also found a new *EIF2AK4* mutation in exon 12 of the patient and his mother. Our results expand our understanding of the *EIF2AK4* mutation spectrum in patients with PVOD, as well as highlight the clinical applicability of WES. More studies should be conducted in the future to explore the influence of the *EIF2AK4* mutation on the development of PVOD and the effect of targeted drug therapy.

## Data Availability

The raw sequence data reported in this paper have been deposited in the Genome Sequence Archive (Genomics, Proteomics & Bioinformatics 2021) in National Genomics Data Center (Nucleic Acids Res 2022), China National Center for Bioinformation/Beijing Institute of Genomics, Chinese Academy of Sciences (GSA-Human: HRA003435) that are publicly accessible at https://ngdc.cncb.ac.cn/gsa-human [12, 13].

## Abbreviations

NGS: Next-generation sequencing
PAH: Pulmonary arterial hypertension
PVOD: Pulmonary veno-occlusive disease
WES: Whole exome sequencing
